# A photovoice assessment for illuminating the role of inland fisheries to livelihoods and the local challenges experienced through the lens of fishers in a climate-driven lake of Malawi

**DOI:** 10.1007/s13280-021-01583-1

**Published:** 2021-06-25

**Authors:** Fiona Armstrong Simmance, Alison Budden Simmance, Jeppe Kolding, Kate Schreckenberg, Emma Tompkins, Guy Poppy, Joseph Nagoli

**Affiliations:** 1 WorldFish, Jalan Batu Maung, Batu Maung, 11960 Bayan Lepas, Pulau Pinang Malaysia; 2 University of Southampton, University Road, Southampton, SO17 1BJ UK; 3 University of Bergen, Postboks 7803, 5020 Bergen, Norway; 4 King’s College London, Strand, London, WC2R 2LS UK; 5 Garden Court Office Park, Area 11, Office Block 3, P. O. Box 30294, Capital City, Lilongwe, Malawi

**Keywords:** Climate change, Food and nutrition security, Inland small-scale fisheries, Lake Chilwa, Livelihoods, Photovoice

## Abstract

Small-scale inland capture fisheries provide an important source of nutritious food, employment and income to millions of people in developing countries, particularly in rural environments where limited alternatives exist. However, the sector is one of most under-valued fisheries sectors and is increasingly experiencing environmental change. This study adopts a Sustainable Livelihoods Approach and investigates how important a fluctuating inland fishery is to livelihoods, and how local perceptions on challenges corresponds to global evidence. Through an innovative participatory method; photovoice, the lived experiences and perceptions of fishers are depicted. The findings illuminate the valuable role of the sector to food and nutrition security and the complex nexus with vulnerability to climate change. The study responds to the call for more local level assessments of the impacts of climate change on inland fisheries in data-limited environments, and the value of the sector in underpinning the Sustainable Development Goals.

**Supplementary Information:**

The online version contains supplementary material available at 10.1007/s13280-021-01583-1.

## Introduction

Wild fish harvested from inland waters, such as lakes and rivers, have an important role to play in food and nutrition security and sustainable development (Funge-Smith 2018; Halpern et al. 2019). Although covering less than 1% of the global water surface, inland fisheries contribute around 25% to global fish supply (Funge-Smith and Bennett 2019) and the sector is still growing (Lynch et al. 2016; Kolding et al. 2019). In vulnerable regions, such as in low-income food deficit countries (LIFDCs), the sector provides a critical source of nutrition and employment where access to quality food and income is limited (Funge-Smith 2018; FAO 2020). Fish can contribute to food and nutrition security through a myriad of pathways. As food, fish is rich in micronutrients, such as vitamin A and iron, and is often one of the most accessible animal source foods for vulnerable rural populations who lack access to formal markets (HLPE 2014). Evidence has shown multiple nutrition and health benefits in consuming fish, including reduced stunting rates in children (HLPE 2014; Headey et al. 2018; Marinda et al. 2018). Livelihoods underpin food security and are the means through which people can economically and physically access food (Connolly-Boutin and Smit 2016; FAO et al. 2020a, b). Inland fisheries provide employment to over 60 million people in low-income countries and contribute substantially to rural economies (FAO et al. 2020a, b). Fish from inland fisheries can act like a cash crop generating employment along its supply chain and providing income which can increase the economic status of households and the purchasing power for food (Kawarazuka and Béné 2010). Inland fisheries can therefore be an important sector in vulnerable regions for reducing poverty and food and nutrition insecurity; offering great potential for underpinning progress towards the Sustainable Development 2030 Agenda and its goals (SDGs) (Funge-Smith 2018; Halpern et al. 2019). Growing research reveals that inland fisheries make substantial contributions towards achieving multiple SDGs, including No Poverty (SDG 1) and Zero Hunger (SDG 2) (Government of Malawi 2017; Lynch et al. 2020). Despite the importance of the sector, it remains one of the most under-reported (by as much as 50–70%) food sectors due to challenges in monitoring, and national statistics mask the importance to sub-sets of populations (Funge-Smith 2018; Halpern et al. 2019). As a result the sector is under-valued and overlooked in management and policies; being largely invisible in the 169 SDG indicators; with no representation in SDG 14: Life Below Water targets, and only one reference in SDG 15: Life on Land (WWF 2021). At the same time, freshwater environments are one of the most vulnerable habitats globally, with over 90% at above-average stress levels and one-third of fish species facing extinction (WWF 2021). Inland fisheries experience multiple threats, such as climate shocks, altered water flows, infrastructure, land-use change, overfishing and pollution (Jul-Larsen et al. 2002; WWF 2021) that can cause a decline in fish supply and irreplaceable loss of nutrients and income. Threats are often outside the fishery, however, the impacts on the sector in low-income countries where data can be limited is not fully known (Kao et al. 2020). In East and Southern Africa inland fisheries are often the main domestic supply of fish with many fisheries driven by climate variability (Jul-Larsen et al. 2002; Kolding et al. 2016a, b). In these contexts, inland fisheries can provide critical benefits to local rural populations (Kakwasha et al. 2020); providing relatively high income compared to agriculture (Ellis and Bahiigwa 2003; Béné et al. 2016), improving dietary diversity (O’Meara et al. 2021) and strengthening resilience (Allison and Mvula 2002). However, some countries such as Malawi experience some of the highest variability in production. The role of inland fisheries to livelihoods and the drivers impacting upon the sector are context specific (Cinner et al. 2010; Kawarazuka et al. 2017), and the contribution of dynamic fisheries to sustainable development in vulnerable regions is still not well understood (Béné et al. 2016; Bennett et al. 2018; Fluet-Chouinard et al. 2018). Furthermore, enhanced recognition and integration of the true value of inland fisheries into development programmes and policy decisions have been called for to better position governments to achieve the SDGs (Diz et al. 2019; Lynch et al. 2020; Halkos and Gkampoura 2021; WWF 2021).

Participatory research has been highlighted as an effective approach to capture the complexity of local context specific factors, to integrate the views and realities of fishers, and to understand the value of and drivers impacting fisheries (Barclay et al. 2017; Bennett et al. 2017). Photovoice is an innovative community-based participatory research method that has been increasingly reported to capture fishers’ and their families personal perspectives through real-life imagery which can generate rich context-specific local knowledge in data-limited environments (Simmance et al. 2016; Pierce 2020). In this paper, we seek to examine the perceptions and lived experiences of fishers through a case study of a climate-sensitive inland fishery in Malawi; Lake Chilwa. A photovoice assessment is applied for the first time in the context of inland fisheries to specifically investigate: (1) How important is a fluctuating, climate-driven, inland fishery viewed for livelihood activity, income, and food and nutrition security for the riparian communities? (2) How and where do the reported global challenges in a climate-driven inland fishery correspond with the stakeholder’s own local perceptions? The Sustainable Livelihoods Framework is adopted which readily describes fishers’ livelihoods within vulnerability contexts, and the range of strategies, capabilities and outcomes achieved.

## Materials and methods

Although a range of qualitative and quantitative methods have been applied in fisheries research, more flexible and creative tools have been called for to (a) capture the complexity of local contexts, including gender (Harper et al. 2013; Kleiber et al. 2015; Bennett et al. 2018); (b) produce policy relevant results (Béné et al. 2016); and (c) to integrate the views and lived experiences of fishers within the management process (Barclay et al. 2017). Participatory research is an effective approach to capture the realities and local knowledge of fishers, for a deeper understanding of vulnerability contexts and livelihoods (Barclay et al. 2017; Rassweiler et al. 2020). One participatory research method; photovoice, has emerged as an approach to understand complex socio-ecological contexts by capturing unique perspectives of marginalised populations in a culturally appropriate and empowering manner (Wang and Burris 1997; Simmance et al. 2016; Pierce 2020). The photovoice process involves providing participants with the opportunity to take photographs of a particular self-chosen issue that are then used to facilitate critical reflection and form a narrative. Throughout the process, participants have control over what they document, what conclusions to report, and how to catalyse change in their communities. The method builds on early livelihoods research by Chambers and colleagues on the ‘Voices of the poor: crying out for change’ which highlighted the value of people’s voice in understanding poverty, challenges and aspirations for improving sustainable development (Narayan et al. 2000). The method has been shown to capture richer and more policy relevant research above traditional methods (Kong et al. 2015), however, its use in fisheries research has been limited (Bennett and Dearden 2013).

**Fig. 1:**
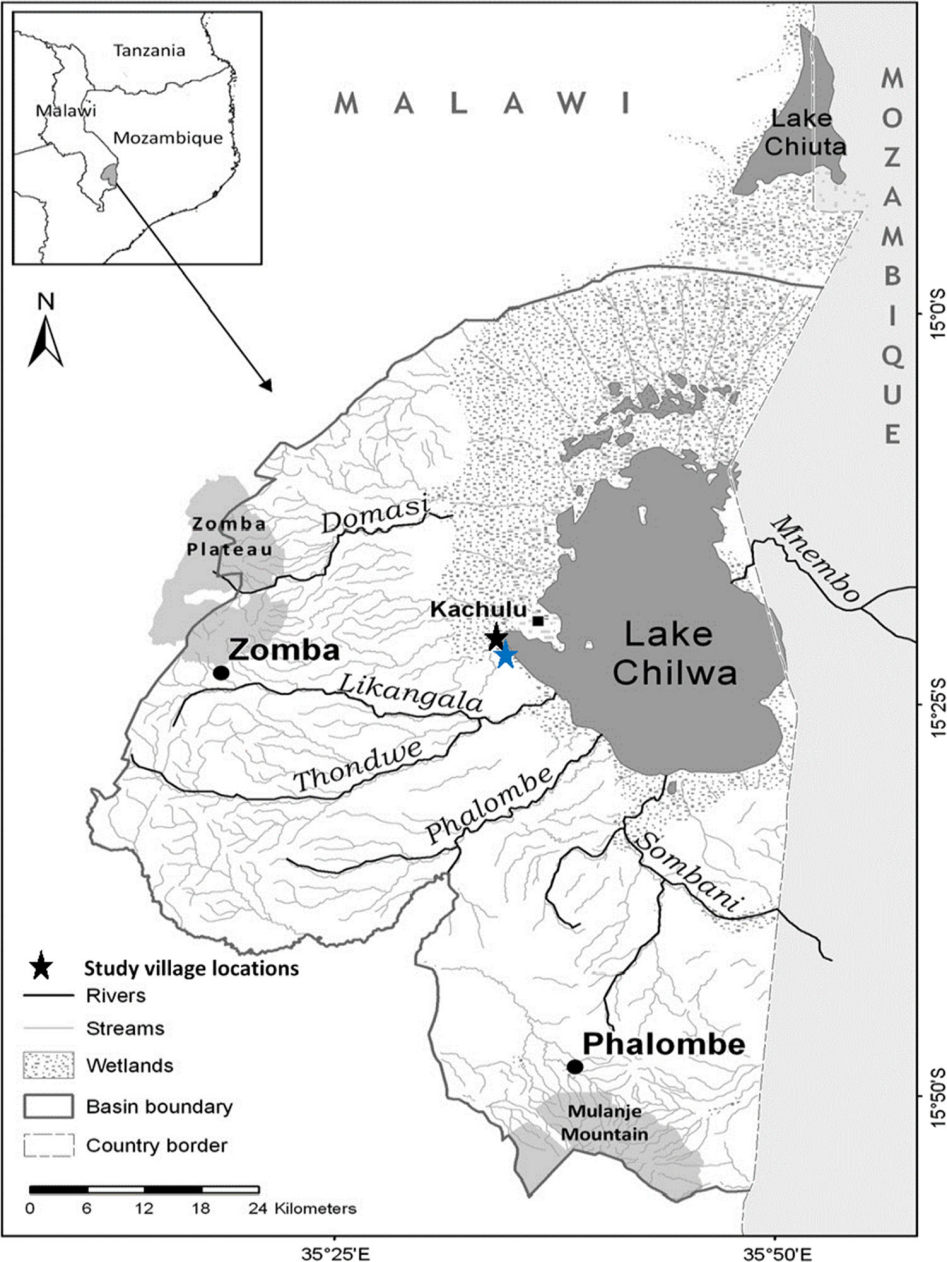
Map of Lake Chilwa in Malawi with study villages (indicated with a star: black representing Village A, and blue Village B) Source: adapted from (Njaya et al. 2011)

Photovoice was applied in two rural communities around Lake Chilwa in southern Malawi (Figs. 1 and 6), in East and Southern Africa. Lake Chilwa is the second largest lake in Malawi with its wetlands designated a Ramsar site of international importance for biodiversity. It represents a typical shallow tropical lake system, with fish production driven by variability in precipitation, and is one of the most productive lake fisheries in Africa but also one of the most unpredictable following short and long-term climate changes (Jul-Larsen et al. 2002). Within the lake’s catchment, thousands of people depend on its natural resources, including fisheries, for their livelihoods and food and nutrition security, where people often adopt mixed livelihoods of fisher-farmers (Allison and Mvula 2002). A fisheries co-management regime operates on the lake to protect the fishery from over exploitation, which implements a closed fishing season from December to March and restrictions on high technology gears (Njaya et al. 2011). The effects of the co-management regulations, which are mainly of the top-down consulting type (Normann et al. 1998), on the ecosystem and livelihoods remain poorly understood (Jul-Larsen et al. 2002; Njaya et al. 2011). At the national level Malawi is one of the member states that adopted the 2030 Sustainable Development Agenda and is committed to implement and report on the progress of the SDGs through the third Malawi Growth and Development Strategy 2017–2022 (MGDS III) (Government of Malawi 2017). As stated in Malawi’s most recent SDG voluntary national review report “the principle of ‘Leaving No One Behind’ stands front and centre in Malawi’s drive to bring the fruits of development to all Malawians, whether child, woman and man regardless of geographical location” (Government of Malawi 2020). However, Malawi is experiencing challenges in long-term progress on almost half of the SDG indicators, including SDG 1 (Eradicating Poverty and ending all its forms).

**Fig. 2:**
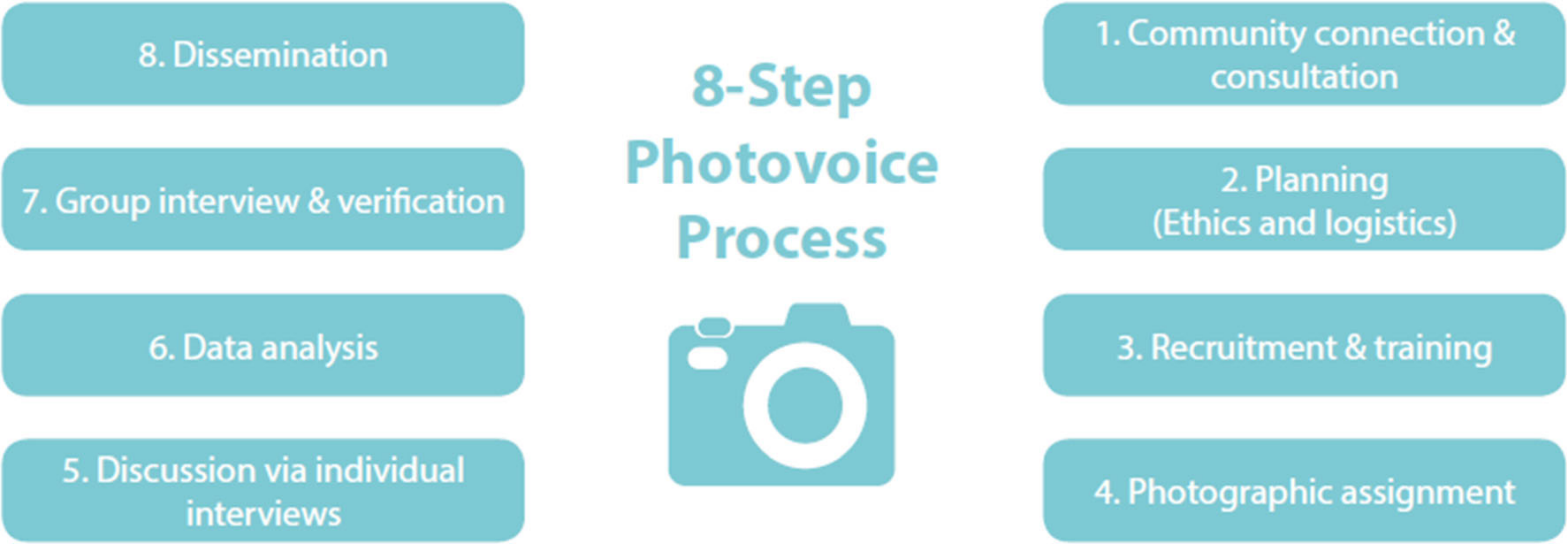
The 8-step photovoice process (Simmance et al. 2016)

**Fig. 3:**
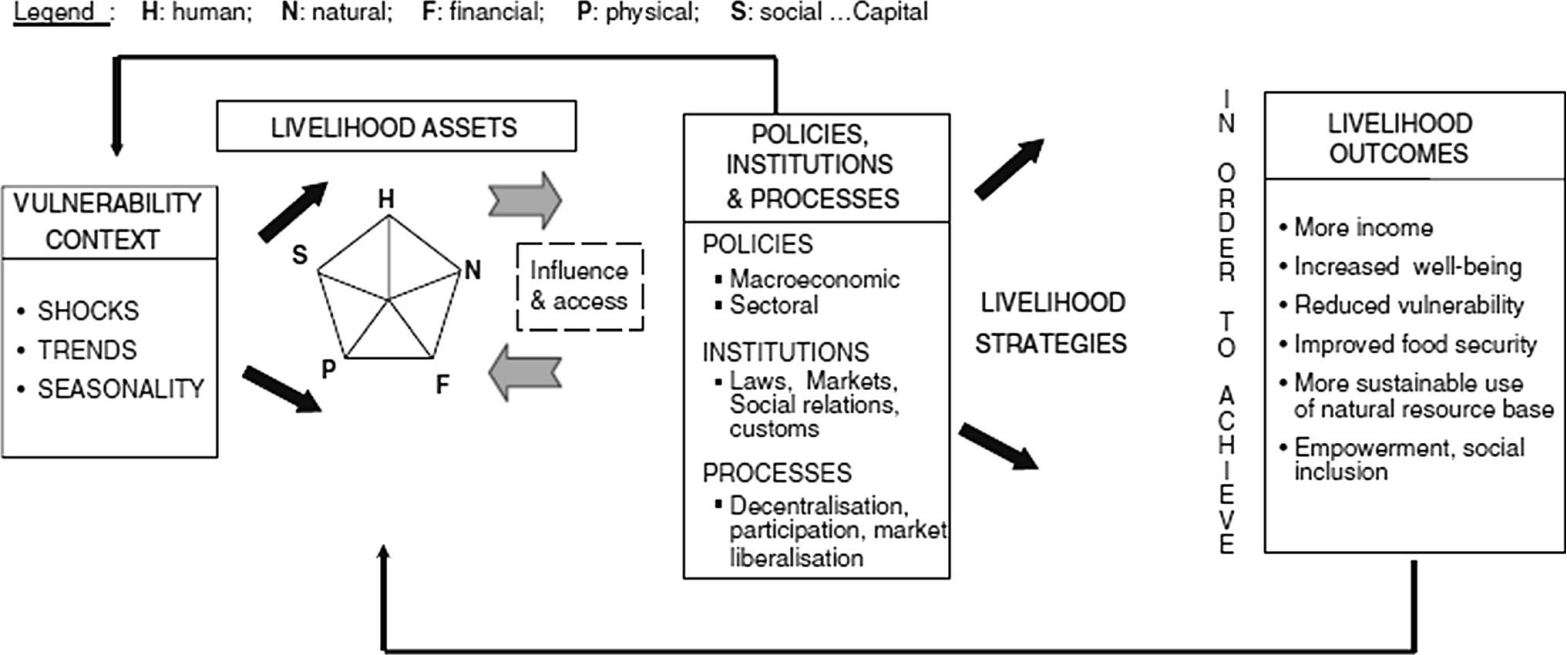
The sustainable livelihoods framework Source: (Allison and Horemans 2006)

**Fig. 4:**
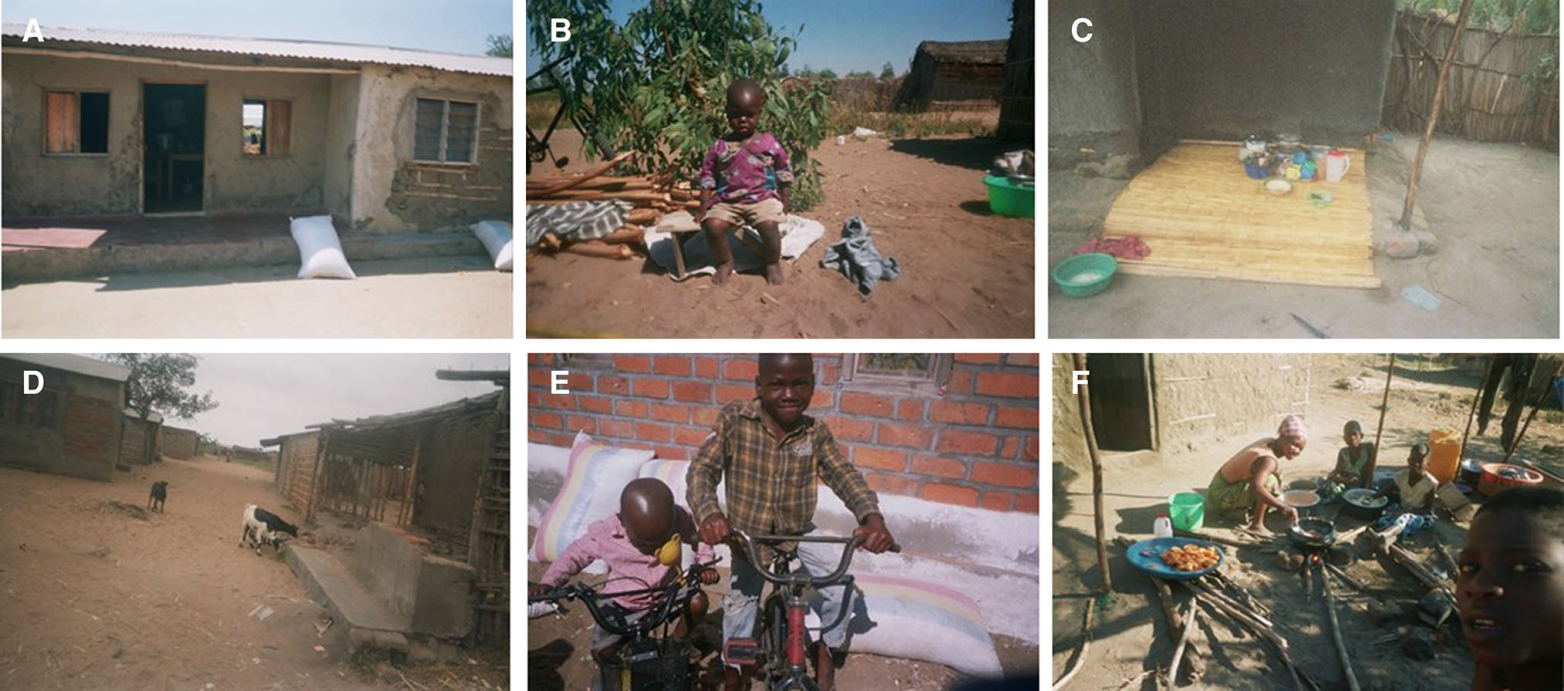
Pictures portraying benefits and livelihood outcomes arising from fishing activities taken by photovoice participants. Moving clockwise from top left corner: **A** House; **B** Clothes for children; **C** Household kitchen utensils and land; **D** Livestock goats; **E** Supporting family with rice for food and bicycles; and, **F** Diversifying livelihood with petty business of samosa selling. Photos by research participants with permission for use obtained

**Fig. 5:**
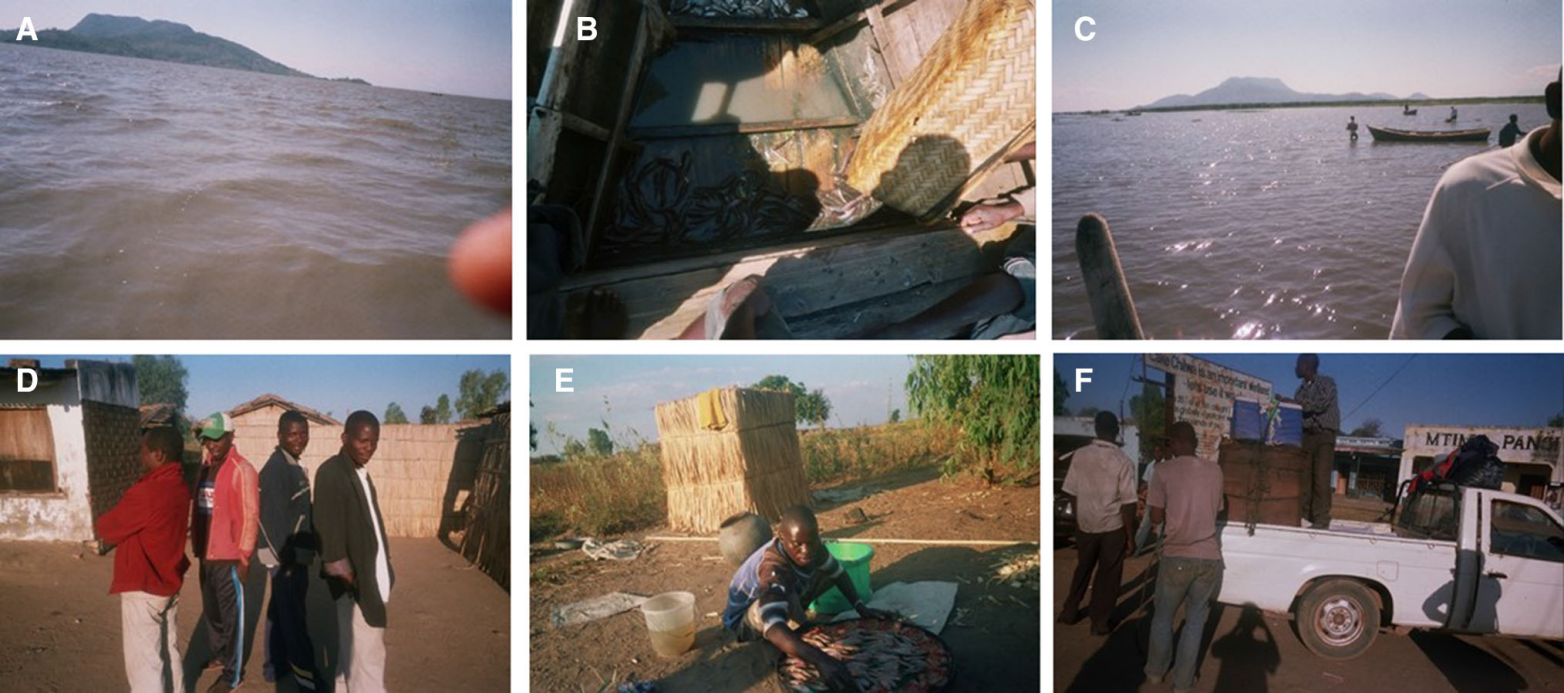
Pictures portraying challenges and the vulnerability contexts in Lake Chilwa’s fisheries taken by photovoice participants. Moving clockwise from top left corner: **A** Wind on the lake in June 2015 effecting fish catches; **B** Scarcity of fish as a result of water levels declining with small amounts of Chambo and Mlamba, and no Matemba species; **C** Low lake levels showing fishermen in waist high level of lake water in July 2015 effecting fish catches; **D** Governance disagreement and a divide between fisheries managers (in red attire) and fishermen; **E** Equipment challenges of availability and renting; and, **F** Transport issues of overcrowding of packages of fish that causes damages and fish losses at market. Photos by research participants with permission for use obtained

**Fig. 6:**
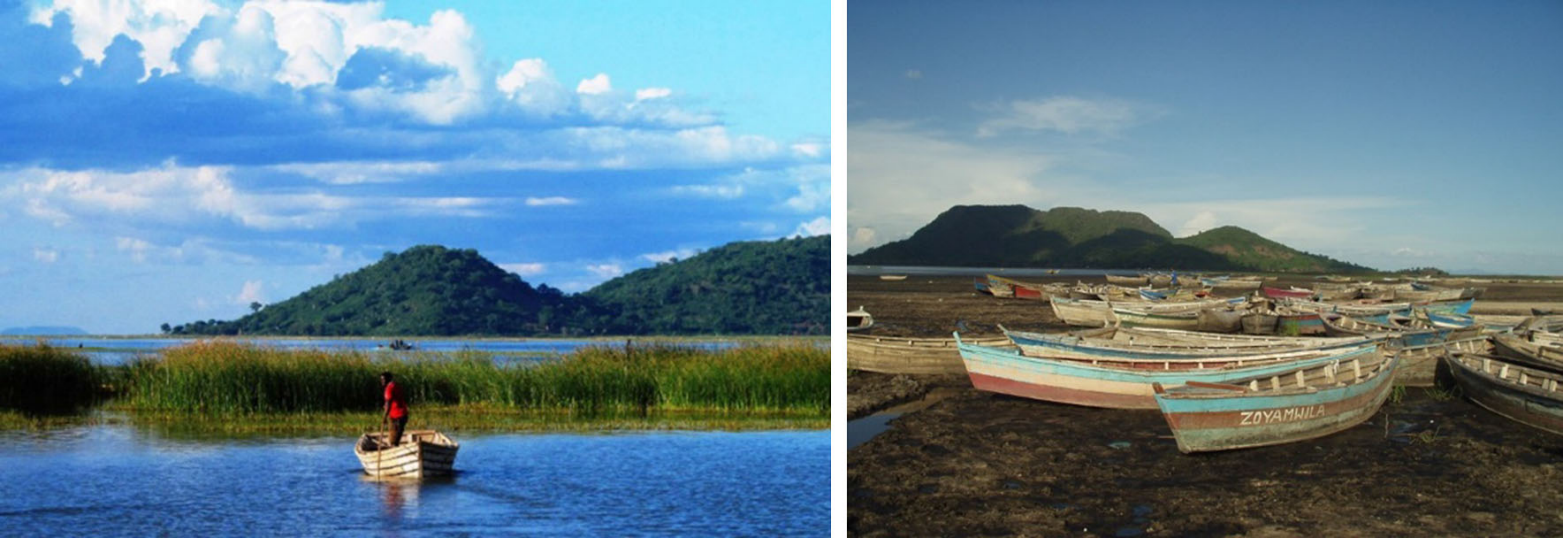
Pictures of Lake Chilwa. Photos by Fiona Armstrong Simmance

The eight step photovoice methodology designed for the context of fisheries and aquaculture by Simmance et al. (2016) was adopted (Fig. 2) (Wang and Burris 1997). Ethical clearance was obtained from the University of Southampton (reference: 14728), the Government of Malawi (National Commission for Science and Technology) and local village leaders prior to fieldwork. The study was conducted between June and August in 2015 in two rural lakeshore villages (A and B, Fig. 1) that represent lakeshore fishing communities. Village A was located next to a road and fisheries landing site; representing greater access to the lake, markets and infrastructure for trade. Village B was located further away (~ 2 km) within the floodplain with access to road and market only by foot which can alter during the wet season. As a rule of thumb, Wang and Burris (1997) recommend to recruit a group of 7 to 10 people to participate in the photovoice method via a combination of snowball and purposive sampling. In line with growing photovoice best practices (Wang and Burris 1997; Hergenrather et al. 2009; Simmance et al. 2016; Suprapto et al. 2020), a total of 15 participants were recruited via purposive and snowballing sampling techniques. A key informant was used in each village to recruit participants based on the following criteria; actively engaged in fisheries in the past 12 months, represent diversity of activities (fish harvester, processor, and trader), and inclusive of men and women. In village A, three men and four women fishers were selected to participate, and in village B, four men and four women fishers were selected to participate (Table 1). Informed consent was obtained from participants prior to the photo assessment and training conducted based on the photovoice field manual developed as part of the study (S1 Photovoice Manual). Each participant was given a disposable camera for one week and asked to take pictures on two topics:

1. What benefits do you receive from fisheries?
2. What challenges do you experience in fisheries?

The topics are open-ended to enable an unbiased exploration on the perceptions of the importance of inland fisheries to livelihoods in terms of achievement of positive livelihood outcomes, and the challenges and vulnerability contexts (Bennett and Dearden 2013). Participants were then asked to select up to 15 photographs that best represented the topics. One to one interviews were then undertaken on the photographs, with the same line of questioning asked and repeated for each photograph:

1. What’s in the picture?
2. Why did you take the picture for that topic?
3. Why did you select this picture over the others?
4. What would you like to tell to others with this picture?
5. Why would it be important to give this message to others?
6. Is there any other information you were unable to capture during the exercise that you would like to share in relation to this topic?

The process is based on the principles of photovoice and promotes Freire’s (1970) concept of critical consciousness through participant critical reflection and dialogue (Wang and Burris 1997). Audio recordings were transcribed and translated, and then analysed via deductive and inductive coding, with interview notes used also for verification. The commonly used three staged process of participatory analysis as recommended by Simmance et al. (2016) (S1 Photovoice Manual) and Wang et al. (1997) was followed: (1) Selecting photographs for discussion; (2) Contextualizing and storytelling; and, (3) Codifying issues, themes or theories with verification with participants individually and as a group.

The Sustainable Livelihoods Approach (SLA) and its framework (Fig. 3) aims to holistically conceptualise the diverse ways that people make a living and can readily describe fisher livelihoods (Allison and Horemans 2006; Scoones 2009). The framework encompasses five dimensions: (1) vulnerability context- trends, shocks, seasonality, and other factors that affect livelihood sustainability; (2) assets—the availability of a portfolio of five forms of capital assets: human, natural, social, financial, and physical, that are the building blocks of livelihoods; (3) transforming structures and processes—institutions and organisations that influence access to assets; (4) livelihood strategies—combination of activities (e.g. fisher-farmer) and choices made in pursuit of livelihoods; and, (5) livelihood outcomes—including changes in human well-being, income, vulnerability, and food security (DFID 2004). The approach and its framework is prominent in a number of fields, including: poverty reduction, food security, climate change and fisheries (DFID 2004; Allison and Horemans 2006; Connolly-Boutin and Smit 2016). Moreover, the framework encompasses the economic, social, and environmental aspects, which are central to the SDGs (Zhao et al. 2019). The framework was used to guide the interpretation of results and discussion.

## Results

All participants completed the photovoice process, with a total of 143 photographs with accompanying narratives analysed (S1 Photovoice Manual). Participants were engaged in a range of fish-related activities with an average of 10 years of experience (Table 1). Men predominately engaged in harvesting of fish whilst women participants predominately undertook fish processing; reflecting the often gendered norms on fish-related activities in the region. Participants expressed a range of benefits in terms of livelihood outcomes, as well as challenges and the vulnerability contexts relating to inland fisheries as discussed in detail below.

**Table 1:** Characteristics of participants

Participant	Age	Sex	Role	Years’ of Experience	Participant Code
Village A					
1	30	Female	Fisher	7	A1_F_F
2	32	Female	Processor	6	A2_F_P
3	32	Female	Processor	6	A3_F_P
4	30	Female	Trader	5	A4_F_T
5	40	Male	Processor	15	A5_M_P
6	40	Male	Trader	4	A6_M_T
7	46	Male	Fisher	29	A7_M_F
Village B					
1	36	Male	Fisher	7	B1_M_F
2	34	Male	Fisher	12	B2_M_F
3	35	Male	Processor	5	B3_M_P
4	61	Male	Fisher	4	B4_M_F
5	28	Female	Processor	7	B5_F_P
6	32	Female	Processor	20	B6_F_P
7	52	Female	Fisher	10	B7_F_F
8	35	Female	Processor	14	B8_F_P

### Benefits and livelihood outcomes from fish-related livelihoods

Small-scale inland capture fisheries was found to contribute positively to local livelihoods. Despite challenges in the sector, generally all participants expressed positive views relating to livelihood outcomes achieved: improved income and food security, reduced vulnerability, and wellbeing (Tables 2 and 3). Differences emerged however between men and women, and between locations, revealing the complexity of local-specific contexts and socio-cultural factors in shaping how benefits are derived and utilised from the sector for livelihood outcomes (Fig. 4).

**Table 2:** Research theme definition

Category	Theme	Sub-theme/description
Benefits and Livelihood outcomes	More income	Fish-related income improves household economic status, and the purchasing power for basic needs and standard of living: food, house/shelter, clothes, education, health etc
	Improved food and nutrition security	Fish directly consumed as food, or fish-related income utilised to meet household food needs
	Reduced vulnerability	Acquiring assets through fish-related income: house, land, livestock, electronics etc., of which can be productive and improve capital assets and diversify livelihood activities
	Wellbeing	Subjective and relational: job satisfaction, identity, pride
Challenges and vulnerability context	Environmental trends, shocks and seasonality	Fishery resource trends (e.g. scarce fish availability), seasonality of production of fisheries and causes such as natural shocks (e.g. drought, floods). Natural challenges relating to predation of fish in fish traps by otters
	Economic shocks and seasonality, and lack of financial capital	Fluctuations in fish prices, challenges in buying and selling, and lack of access to financial services (e.g. loans)
	Lack of physical capital	Lack of access to markets, infrastructure and transport for fish trade, as well as technology for effective fish preservation and reductions in fish quality losses and waste. Challenges in access to equipment (e.g. nets, processing racks etc.) in terms of price, servicing and availability
	Policies, institutions and processes	Governance and management of fisheries and challenges in access to resources—rules and regulations in terms of equipment (e.g. fishing gears), fishing rights and temporal utilisation (e.g. closed seasons). As well as labour and rights issues
	Lack of social capital	Security challenges—such as theft of equipment
	Lack of human capital	Health—general poor health concerns of individuals, and from fish-related activities (e.g. smoking fish)

**Table 3:** Selected quotations from participants representing benefits and livelihood outcomes from their fish-related livelihood activities

Theme	Quotations from participants
Overall benefits of the sector	“In fishing we have challenges but the benefits surpass the challenges” (Participant A1_F_F. Figure 4A)
Improved food and nutrition security	“Without fishing I would not have been able to buy clothes for my family and food to support them”. (Participant A1_F_F, a female fisher. Figure 4A)
More income—basic needs—clothes	“Out of my business I have been able to buy some clothes for my child” (Participant B5_F_P, a female processor. Figure 4B)
More income—human capital—education	“With my fishing business I have been able to provide all the needs for my children’s education” (Participant A6_M_T, a male trader)
More Income—physical and natural capita—land, house and kitchen utensils.	“Indeed this business has been so profitable to me [fish processing and selling]…I never anticipated that one day I would have my own house, have kitchen utensils and own piece of land, but it is all out of this business”. (Participant A2_F_P, a female processor. Figure 4C)
Reduced vulnerability—physical assets—house	“It is not a very hard thing for one to have a house with iron sheets roof. All it matters is that one should have hands. From the fish we take for granted one can have a house with iron sheets like myself” (Participant B8_F_P, a female processor. Figure 4D) “To build a house it is a very big thing that’s why I took a picture because it is the very first thing that came from the first profits we made from fishing” (Participant A1_F_F, a female fisher)
Reduced vulnerability—physical assets—bicycle	“When I started processing and selling fish, one of the very first things that I bought was the bicycle and the rest of the things I bought later….as I noticed that it was difficult for me to transport and walk from where I am to smoke [fish] and to the market so I wanted my own mode of transport….the bicycle enabled me to earn more and more money” (Participant B3_M_P, a male processor)
Reduced vulnerability—diversifying livelihood activities	“Out of fish processing I was able to construct a house and buy farm animals and have goats… this helps me a lot in the way that sometimes I use them for food, and at other times I can sell some and use the money to pay school fees for my children.” (A5_M_P, a male processor. Figure 4E)
	“Having two businesses is good because at times it may happen that there is no fish to buy at the port because it was windy on that day, so I use the money which I earn from the samosa selling business” (Participant B6_F_P, a female processor. Figure 4F)
Wellbeing	“A woman should not take herself as a failure. It is possible for a woman to go to the lake, buy fish, process it and from then be able to sustain herself….I am advising as well as encouraging women” (Participant B8_F_P, a female processor. Figure 4A) “You too should work hard to realise the benefits like the ones I have been able to realise” (Participant A2_F_P, a female processor. Figure 4C)

#### More income and improved food security

The majority of participants (*n* = 12) outlined that through their fish-related livelihood activities, they were able to meet their household needs specifically in relation to improving food and nutrition security through direct (fish as food) and indirect (fish as income) pathways (Fig. 4E). A female fisher stated; “In fishing we have challenges but the benefits surpass the challenges…without fishing I would not have been able to buy clothes for my family and food to support them” (Participant A1_F_F. Figure 4A). Some participants (*n* = 4) also discussed the multiple benefits gained from having more income because of their fish-related activities, such as improved human capital, where male fishers in particular outlined that with their fish-related income they were able to pay for their children’s education; *“With my fishing business I have been able to provide all the needs for my children’s education” (Participant A6_M_T, a male trader).*

#### Reduced vulnerability

In addition to meeting the basic needs of the household, most fishers (*n* = 13) also highlighted the value of fish-related income in improving the financial capital of the household and reducing vulnerability. Participants explained that their fish-related income enabled them to acquire assets that were very important for sustaining their household needs and for coping with and adapting to challenges. A range of assets were outlined which included basic needs such as clothes, household goods (e.g. for cooking) and electronics (e.g. phone) (Fig. 4B). Productive assets relating to physical capital: house, bicycle, fish-related equipment, were acquired which provided security and the tools needed to strengthen livelihood activities (Fig. 4C). Participants also invested in natural capital: land, farming crops and livestock rearing, as well as businesses (e.g. small shop) and petty business (e.g. selling a range of foods) to diversify livelihood activities (Fig. 4D and F). Differences emerged between locations and men and women on the types of assets acquired. In the market connected village (A), several fishers outlined that their fish-related income enabled them to construct their own house (*n* = 4) and acquire small businesses such as a shop (*n* = 3). Fishers expressed immense pride in being able to own their own home and ranked it as a top livelihood outcome from their inland fisheries livelihood; *“It is not a very hard thing for one to have a house with iron sheets roof. All it matters is that one should have hands. From the fish we take for granted one can have a house with iron sheets like myself” (Participant B8_F_P, a female processor. Figure* 4*D)*. In the more remote village (B) on the other hand, participants discussed purchasing a bicycle (*n* = 4) out of their fish-related income to improve trade and market access (Table 3). Male fishers (*n* = 4) more frequently outlined investment of fish-related income into livestock as a livelihood strategy, whereas female fishers (*n* = 3) predominately discussed purchasing household goods and clothes more often, and investment in petty businesses to diversity livelihoods (Table 3).

#### Increased well-being

Fishers also obtained wider dimensions of wellbeing from their fish-related activities; including individual pride in their activities, self-actualisation, independence, strong identity and job satisfaction, which was particularly portrayed by women fishers; *“A woman should not take herself as a failure. It is possible for a woman to go to the lake, buy fish, process it and from then be able to sustain herself….I am advising as well as encouraging women” (Participant B8_F_P, a female processor.* *Figure* 4*A)*. In addition, fishers highlighted the pride and value of working hard to achieve benefits from the sector.

### Challenges experienced and the vulnerability contexts of fish-related livelihoods

The vulnerability contexts portrayed centred on three components of livelihoods: environmental shocks, trends and seasonality of fish resources; economic shocks and lack of access to financial capital in terms of loans; and policies, institutions and processes in terms of governance of fisheries and access (Fig. 5, Table 2). Similarities were found between men and women participants as well as between locations (Table 4).

**Table 4:** Selected quotations from participants representing challenges experienced in their fish-related livelihood activities

Theme	Quotations from participants
Environmental seasonality and fish resource availability	“When there is wind blowing on the lake they should not go to fishing…because it’s too dangerous….and they will not catch anything” (Participant A7_M_F, a male fisher. Figure 5A) “When it is very windy the water becomes muddy and the fish do not swim anymore they just hide somewhere so when it is windy it is always hard to catch any fish” (Participant B2_M_F, a male fisher)
Environmental shocks and fish resource availability	“In a year, it is especially June and July when fish is scarce, but, recently this has been a problem due to water levels coming down, the lake has been drying. Matemba has become completely scarce now. At least Mlamba and Chambo can catch in little amounts” (Participant B1_M_F, a male fisher. Figure 5B) “Lake Chilwa nowadays is no longer able to give us a considerable large amount of fish, particularly Matemba, when compared to the past. Because in the past, it was the case that fishermen could just throw away Matemba because there was too many, but these days one cannot do that…Due to water levels coming down, the lake has been drying” (Participant A2_F_P, a female processor)
Environmental trends and fish resource availability	“Our lake, Lake Chilwa, does not have enough water…normally, it would not be possible for people to be walking on the lake, but here we can see that people can walk just like that even without using boats” (Participant B1_M_F, a male fisher. Figure 5C) “In the future the amount of fish will be increasing, also especially Chambo and Matemba will start multiplying in large numbers again, because, now water levels are rising again.” (Participant A2_F_P, a female processor)
Environmental seasonality and impacts on livelihoods	“When it is windy, fishermen are not able to catch a lot of fish, now this becomes a problem even at the household, because people are not able to have relish for the day” (Participant B7_F_F, a female fisher)
Environmental trends and coping strategies	“A word of advice to my colleagues, sometimes relying on Lake Chilwa for business is risky, as sometimes it becomes hard, sometimes you cannot find Matemba or the fish species we were expecting, but they should think of having a variety of selling items for them to be safe” (Participant A2_F_P, a female processor)
Economic shocks	“When there is drought fish is scarce and it is expensive to buy and in such cases we make losses when we sell it” (B8_F_P, a female processor)
Policies, institutions and processes—fisheries governance	“There is no agreement, no oneness between the two groups… instead of simply advising the fishermen never to use the nkhoka net during the closed season, they instead hide and wait for them to go and do their fishing then arrest them and ask them to pay the fine, which they do merely to get the money for themselves” (Participant B1_M_F, a male fisher. Figure 5D)
Lack of physical capital	“As a fish processor, I encounter a number of problems, one of them is that sometimes I am not able to rent a tin for smoking the fish, so I end up creating a hole on the ground so that I smoke the fish instead of letting the fish get rotten” (Participant B5_F_P, a female processor. Figure 5E) “There is also transport problems as we want to sell fish to another area… when we are going to the market the fish get damaged on the way due to over packing of things and they also get dirty” (Participant A6_M_T, a male trader. Figure 5F)

#### Environmental trends, shocks and seasonality

Poor fish availability was the most frequently (*n* = 13) noted challenge by fishers caused by seasonality and unforeseen shocks in climate. Most of the participants (*n* = 13) discussed the effect of seasonal wind patterns; known locally as ‘Mwera’ winds, on the availability of fish each year during the months of May to July, with a few also describing the cold temperatures during similar months as impacting on fish availability. Participants described how the winds affected the catchability of fish and the safety of fishermen where some fishers lose their lives; *“When there is wind blowing on the lake, they should not go to fishing…because it’s too dangerous….and they will not catch anything” (Participant A7_M_F, a male fisher.* *Figure* 5*A).*

The long-term trends in fish availability were also discussed by fishers (*n* = 10) specifically in relation to the three main fish species caught in Lake Chilwa. Drought and receding water levels over the past few years was outlined by many to impact upon the availability of Matemba (*Barbus paludinosus*), followed by the endemic Chambo (*Oreochromis Shiranus chilwae.* Also known as Makumba), with little impact on the catfish—Mlamba (*Clarius gariepinus*); *“recently this has been a problem due to water levels coming down, the lake has been drying…Matemba has become completely scarce now” (Participant B1_M_F, a male fisher. Figure *5*B*). A few participants also discussed the dynamics of the lake level and its impact on fish availability in relation to a flooding event a few months earlier in January and February (2015) to the study period. One participant felt positive about the increased rainfall and rise in lake levels having a potential positive impact on fish availability in the future, whilst another stated that concerns on fish availability and drought remained, as the most recent rainfall event was not sufficient to sustain water levels (Table 4, Fig. 5C).

A deeper perspective on the impact of fish availability on fisher’s livelihoods was outlined by some participants detailing the impacts over the short and long term. One female fisher outlined the immediate effect of seasonality and winds causing low fish availability on day-to-day household food security. A few fishers also discussed the longer-term impact of drought-induced fish scarcity on their livelihoods, where fishers had to sell personal assets to cope with reduced income. Participants outlined diversifying livelihoods, such as through livestock and other businesses, were essential to coping with and adapting to the challenges in the sector and reducing vulnerability. A female processor outlined advice to other fishers *“A word of advice to my colleagues, sometimes relying on Lake Chilwa for business is risky, as sometimes it becomes hard, sometimes you cannot find Matemba or the fish species we were expecting, but they should think of having a variety of selling items for them to be safe” (Participant A2_F_P).* A small number of fishers also provided suggestions to adapt to future shocks in the fishery, such as diversifying their livelihood activities into aquaculture.

#### Economic shocks, lack of financial and physical capital

Participants (*n* = 10) described economic challenges which included lack of profits, fluctuations in prices, difficulty in buying and selling fish, and lack of loans and credit unions. Many outlined the difficulty in taking pictures of these challenges and therefore resorted to expressing them during discussions. Fishers most frequently discussed lack of profits as a constraint, in particular by fishers from village B. Several participants linked lack of profits with environmental shocks and the impacts on fish availability causing fluctuations in prices; *“When there is drought fish is scarce and it is expensive to buy and in such cases we make losses when we sell it” (B8_F_P, a female processor).* A few fishers from village A also mentioned the challenge of having no financial loan institutions to enable them to grow their businesses, particularly during the past few years during low lake water levels and fish scarcity. Fishers also discussed challenges with access to physical capital such as markets, equipment, technology and effective transport storage and preservation to reduce fish quality losses (Fig. 5E and F). Participants (*n* = 6) from the remote village (B) in particular portrayed challenges in acquiring equipment in relation to access, servicing and price, where they are often reliant on outside fish traders for access.

#### Policies, institutions and processes

Governance issues was also expressed by a few fishers (*n* = 6). Participants discussed disagreement with the top-down imposed co-management rules of closed seasons, compliance issues and the impacts on their livelihoods. Participants from both villages stated tension with fisheries managers and the associated Beach Village Committees governing (BVCs), however, fishers from village B outlined challenges of trust and the negative impact of the closed season on livelihoods. One male fisher states *“There is no agreement, no oneness between the two groups” (Participant B1_M_F.* *Figure* 5*D).* Participants also outlined the challenges of compliance with fishing equipment regulations due to the high cost of equipment and access challenges.

## Discussion

Inland fisheries can underpin sustainable development through the multiple economic and nutritional benefits of fish. However, the sector is persistently under-valued and is one of the most threatened environments globally. In vulnerable regions and data-limited environments, such as East and Southern Africa, the contribution of dynamic inland fisheries to livelihoods, and the threats experienced are still not fully known or appreciated. Through capturing the voices of inland fisher communities in a climate-sensitive lake fishery; Lake Chilwa, in Malawi, this paper sheds light on the lived experiences and perceptions on the importance of the sector to livelihoods, and local challenges experienced.

First, photovoice was a useful process for understanding the pathways through which inland fisheries contributes positively to livelihoods, including direct and indirect pathways to improved food security and well-being. Overall, Lake Chilwa’s fluctuating, climate-driven inland fishery was perceived as important for strengthening livelihoods by lakeshore communities. Participants portrayed multiple livelihood benefits of fish-related activities; improved income and food and nutrition security, reduced vulnerability and improved wellbeing, with inland fisheries supporting the basic needs and welfare of lakeshore communities (Allison and Horemans 2006; Scoones 2009). Wider evidence in the region has also found that inland fisheries can contribute substantially to rural household income, often higher than agriculture (Pollnac et al. 2001; Kawarazuka and Béné 2010). Participants also outlined diversified livelihoods, which is common in inland fishing communities and vulnerable regions (Allison and Mvula, 2002; Kolding et al. 2016a, b), and the sequential nature of livelihood strategies where many highlighted that fish-related activities provided the extra income that increased their opportunities to achieve desired livelihood outcomes. In addition, the contribution of fish-related livelihoods to food and nutrition security was shown via direct—fish as food, and indirect—fish as income pathways and purchase of staple foods, which is increasingly being found in other contexts where inland fisheries can increase dietary diversity (Darling 2014; Hartje et al. 2018; Moreau and Garaway 2018; O’Meara et al. 2021). However, there were also differences found in the utilisation of income beyond food security, with participants investing in material and productive assets, such as natural (e.g. livestock and land) and physical (e.g. house) assets to increase wealth and security, and to diversify livelihoods and reduce vulnerability. These findings show that inland fisheries can also contribute to the alleviation of poverty through improved wealth generation and reduced vulnerability; disputing past assumptions that fishers are the poorest of the poor and that fishing is an employment of last resort (Pollnac et al. 2001; Scoones 2009; Béné et al. 2016). However, investment in the types of assets differed by location, showing inequalities in income and access to opportunities between fishing communities. Further research is needed into the access and utilisation of assets, and how they are transformed into positive livelihood outcomes during times of change (Nagoli and Chiwona-Karltun 2017).

Gender is known to have a significant role in determining the different mechanisms and processes that generate livelihood outcomes such as improved food security, wellbeing and increased income (Kawarazuka et al. 2017). Differences were found in how fish-related income was utilised between men and women, which reaffirms the dominant role of women in taking care of household needs (Geheb et al. 2008; Kawarazuka et al. 2017). Further research is needed on women’s empowerment in the sector and also the power relationships and decision-making between men and women in the sector and within households (Manyung-Pasani et al. 2017).

Second, the photovoice process provided new insights into how challenges are experienced and viewed at the local level and the vulnerability contexts of inland fisheries; revealing richer, more unexpected information. Fishers experienced multiple challenges relating to environmental fluctuations, trends and seasonality of fish resources, economic shocks and lack of access to financial capital, and policies, institutions and processes in terms of governance of fisheries, with similarities between men and women fishers (Allison and Horemans 2006; Scoones 2009). The vulnerability context in terms of environmental instabilities and scarcity of fish was the most frequently reported challenge, with reduced rainfall and drought negatively impacting upon fisheries. The finding reaffirms regional evidence of climate being a large driver of fisheries in shallow tropical lakes (Okpara et al. 2016) and the periodic drying and fluctuating fisheries of Lake Chilwa (Jul-Larsen et al. 2002; Njaya et al. 2011; Gownaris et al. 2018). A few participants stated that household assets were sold to cope with the drought-driven scarcity of fish, particularly in years since 2012, revealing the vulnerability of fisher livelihoods to long-term climate changes that can erode the asset base of households. However, as highlighted by Kolding et al. (2016a, b), climate variability can also present opportunities for fisheries, such as increased productivity from water level fluctuations and during periods of good rains. The photovoice process illuminated the value of local knowledge where a few participants expressed the dynamic nature of climate trends and stated that a recent flood could bring new productivity to the fishery over the coming years. More immediate impacts of climate variability were also reported by participants in relation to seasonality and wind patterns affecting freshwater ecosystems, fish catches and livelihoods. This is an important finding as other studies have shown difficulty in understanding seasonality in data-limited environments (Jul-Larsen et al. 2002) as well as understanding wider climate variable impacts (Berbés-Blázquez 2011; Okpara et al. 2016; Kao et al. 2020). The local perceptions on the importance of climate drivers reaffirm global evidence on the role of external drivers impacting inland fisheries, and highlights the complexity of understanding positive and negative impacts of diverse climate factors (Kolding et al. 2016a, b; Gownaris et al. 2018; Kao et al. 2020), including the value of local knowledge. Wider challenges related to economic shocks in fish prices, lack of access to financial capital (e.g. loans) and physical capital (e.g. technology to reduce fish waste and loss) were reported with inequalities between locations. These challenges demonstrate the untapped potential and opportunities of the sector to enhance supply, such as through reductions in waste and loss (Kakwasha et al. 2020; Torell et al. 2020) with improved transport and preservation technology; highlighting the importance of examining internal factors and the challenges and opportunities across value-chains (Darling 2014; Béné et al. 2016). The third challenge perceived by fishers related to policies, institutions and processes in terms of governance of fisheries and access to resources, where closed fishing seasons and gear restrictions were perceived to negatively impact upon livelihoods (Béné et al. 2016). Livelihood challenges in relation to fisheries governance were perceived more severely by participants from the remote village, who portrayed greater challenges in accessing. There was a lack of perceived challenges from wider environmental factors as reported globally; such as excessive water extraction, infrastructure (e.g. dams), land-use change, overfishing and pollution (Jul-Larsen et al. 2002; WWF 2021). Most likely due to the temporal or distant external nature of such threats. The perceived importance and realities experienced by fishers generally portrayed immediate and direct impacts that affected their livelihoods. Our findings highlight that the needs, realities and perceptions of fishers are critical to understand constraints and opportunities for improved livelihoods and management of fisheries, which underpin sustainable development (Bennett and Dearden 2013; Barclay et al. 2017). Recent research has demonstrated the impacts of land-use change in Lake Chilwa’s catchment, with a reduction of 80% of its wetlands over the past few decades due to land cultivation and rice farming, particularly in the north, however, the effects on fisheries are still not known (Pullanikkatil et al. 2020). Further research is needed into the evolving challenges and impacts upon inland fisheries and fishers, such as arising from COVID-19 pandemic, and how experiences differ between actors.

Thirdly, aside from material benefits, fishers also revealed rich information on subjective well-being such as individual pride and satisfaction. Several participants expressed pride in their roles and strong identity linked with hard working, business focused and risk-taking characteristics. Women fishers however expressed more in-depth independence, self-reliance, and empowerment, and were proud that they were able to do male dominated roles. These findings reveal that most fishers perceived their fisher activities not merely as a subsistence livelihood activity but as a career and way of life that enabled them to improve their standard of living. This corroborates similar findings by Weeratunge et al. (2014), and adds a deeper understanding on the perceptions and role of women in the sector.

## Conclusion

Inland capture fisheries provide employment, income, and a nutrient-dense food source to millions of people in developing countries, particularly in rural environments as part of a diversified economy. However, the sector is the most under-valued and neglected food production systems, and its contribution towards food and nutrition security and resilience in vulnerable regions in not fully explored. This study applies a participatory photovoice assessment for the first time in an inland fishery context to investigate how a fluctuating, climate-driven inland fishery is viewed to in relation to livelihoods, and how local perceptions of ambient challenges correspond with the global picture. The experiences and realities of inland fishers in the shallow, fluctuating Lake Chilwa, southern Malawi, are portrayed through their voices and ´real life´ imagery to provide valuable contextualised and varied information in a data-limited environment. Overall fishers perceived fish-related livelihoods as generating positive outcomes relating to improved income, food security and wellbeing, and reduced vulnerability. Fishers also portrayed the vulnerable contexts of inland fisheries, with climate variability a main external threat but also an opportunity, and economic setbacks, lack of physical assets (e.g. technology and access to markets) and inequalities in access, affecting fisheries and livelihoods. The local perceptions of challenges reflected the daily realities and differed from global discourse on the multiple external threats affecting upon the sector. This highlights the importance of accounting for local knowledge, and the needs and realities of fishers for adaptive and contextualized effective management. Our findings provide a case study for illuminating the overlooked importance of inland fisheries for underpinning the Sustainable Development Goals (SDGs), such as reduced poverty (SDG1) and hunger (SDG2). However, only by recognising the realities and needs of fishers within the sector and highlighting the value of inland fisheries to the wider society, will governments, policy makers and managers be better poised to safeguard inland fisheries and progress the SDGs. More participatory research is needed in diverse inland fishery contexts, where photovoice can be used as an effective tool for fishers to share their voices to wider contexts and regions.

## Supplementary Information

Below is the link to the electronic supplementary material.

Electronic supplementary material 1 (PDF 638 kb)
