# Supplementary material for: A photovoice assessment for illuminating the role of inland fisheries to livelihoods and the local challenges experienced through the lens of fishers in a climate-driven lake of Malawi
Source: Ambio. 2021 Jun 25;51(3):700–15. doi: 10.1007/s13280-021-01583-1 (PMC8231085; doi:10.1007/s13280-021-01583-1)
Supplement: Supplementary file 1 — Electronic supplementary material 1 (PDF 638 kb) [file 13280_2021_1583_MOESM1_ESM.pdf]

Ambio

Electronic Supplementary Material

This supplementary material has not been peer reviewed.

Title: **A photovoice assessment for illuminating the role of inland fisheries to livelihoods and the local challenges experienced through the lens of fishers in a climate driven lake of Malawi.**

S1 Photovoice Manual for Fisheries and Aquaculture Research

METHODOLOGY GUIDE FOR FIELD WORK

EIGHT STEP METHODOLOGY:

1. COMMUNITY CONNECTION and CONSULTATION; Building trust.
2. PLANNING; Funding, logistics, ethics.
3. RECRUITMENT and TRAINING Participant identification, introduction, camera distribution and instructions
4. PHOTOGRAPHY ASSIGNMENT and COLLECTION; Periodic check-in on participants, camera collection and development.
5. DISCUSS PHOTOGRAPHS THROUGH INTERVIEWS; Develop narrative, reflect on images.
6. DATA ANALYSIS; Coding of main topics and themes.
7. PRESENTATION OF FINDINGS and DISCUSSION OF OUTCOMES; Cross-check interpretations, discussion on outcomes and implications.
8. DISSEMINATION; Ownership, desired audience- academia, public, policy.

A. PRIOR TO THE EXERCISE (STEPS 1 and 2)

1. Identify case study villages and plan all field logistics accordingly.
2. Introduce the aims of the study to community traditional leaders within the case study villages. Build a level of trust with the community via regular communication and transparency in study aims.
3. Identify adult females actively engaged in capture fisheries from the case study villages.
4. Try to locate fishers from a diverse range of wealth status and years of experience in the fishing sub sector.
5. Provide training to the translator and ensure that he/she is informed of the entire procedure in advance of presenting the exercise.

B. PRESENTING THE EXERCISE (STEPS 3 and 4)

1. Recruit a group of 7 to 10 participants via a combination of snowball and purposive sampling of participants. Invite participants to a training workshop in order to present the project.
2. At the moment of presenting the project discuss the following aspects:
    a) The objectives of the research project.
    b) The parties involved.
    c) Their rights to refuse participation or withdraw from the exercise at any time.
    d) The use we intend to give to the images collected (stress that they are not for commercial use).
    e) Any form of compensation that may be provided (none, on this occasion, except for giving back all pictures they took).
3. Ask participants to sign the consent form (**attached to this document**) or record a verbal agreement with use of a Dictaphone.
4. Explain the following to all participants:
    a) **Time and length of exercise**: For this study, participants will take pictures about the topics detailed below for a total of two weeks. Explain that each participant will be given a camera and that they only have a limited number of photos to take (specify total cap of photos). Each participant is to therefore think carefully about the questions and ensure pictures that are captured cover each topic. A research member will check-in on participants after 1 week to ensure the exercise is progressing fine. A telephone number will also be provided should participants have queries or problems throughout the exercise. Explain that the exercise does not finish when they return the camera but that you will come back to ask a few questions about the pictures she took after the pictures have been taken.
    b) **How to operate the camera**: Use **visual aids** to facilitate instructions of the project. Explain how the camera works. Use a spare camera for this purpose. Make sure participant understands how it works. Remind participants of the following aspects:
        - The camera has up to (specify total number of pictures) takes and that there is a counter available.
        - Explain that the camera does not reload automatically.
        - Show them how the flash works for takes in the dark.
        - Explain that it is waterproof and that participants are encouraged to take photos whilst actively carrying out activities in relation to fishing.
        - Explain that if they want to take pictures of people or specific objects, they should not be too far away or too close (usually in between 2 to 5 meters).
        - Remind them to be careful with mud and dust since the cameras are not rugged.
        - Once you have explained these topics, hand over the spare camera to the participant to show you that she understood.
    c) **Topics**: Tell the participant that we would like her to take pictures about the following three topics below. After you explained the subjects. Ask the participant to take one picture for each topic with the spare camera. Review why they took those pictures so as to make sure they understood the subjects.
        - **WHAT ACTIVITIES** do you carry out in relation to capture fisheries? Ask participants to capture pictures about their day to day involvement in the fishery. Use the visual aid to explain examples of what this might include (see attachments).
        - **WHAT BENEFITS** do you receive from capture fisheries? Explain to participants that they are to capture pictures about benefits arising from their involvement in the fishery. Explain that this may include: increased fish/food for the household, increased income, etc. Ask participants to take pictures about a range of perceived benefits.

- **<u>WHAT CHALLENGES</u>** do you experience in capture fisheries? Ask participants to take photos about challenges experienced in the fishery. Use the visual aid to explain examples of what this might include (see attachments).

d) **<u>Safety considerations when taking pictures:</u>** Discuss the following topics with the participants:
   - Remind participants that the cameras are rather inexpensive and have a single use (i.e., they are disposable). Consequently, the risk of theft is minimal; however, they should ensure that they do not expose it too much to avoid conflicts.

e) **<u>Ethical considerations when taking pictures:</u>** Remind participants that they cannot take pictures of people freely. In particular remind them the following:
   - They can freely take pictures of their family members but if they want to take pictures of other individuals, they should ask for permission.
   - There are no restrictions for taking pictures of objects or landscapes (as long as it is safe).
   - They MUST not take pictures of very ill or very old people. That is, those who cannot refuse to have their pictures taken.
   - They MUST not take pictures of naked people, including children.
   - They MUST not take pictures of people in a compromising situation (e.g., in the toilet, conducting illegal activities or after an accident).

f) Once participants have understood the instructions, **<u>agree on a date and time</u>** suitable for the participant for you to **<u>collect the camera</u>** in two weeks' time. Confirm any necessary telephone contact details should the time/date need to change or the participant has any questions during the exercise (reminding them that a research member to check-in in 1 weeks' time).

g) After the pictures have been revealed at a local camera/printing store, **<u>agree on a date and time</u>** suitable for participants to carry out an **<u>interview about what the photographs</u>** mean to participants.

C. DISCUSSING THE PICTURES (STEP 5)

1. On the interview day you should bring all the pictures that the participants took during the 2 week period.
2. Proceed in the following manner:
   a) Ask the participant to select **<u>ONE</u>** picture that better illustrates the activities she carries out in relation to fishing
   b) Once the picture has been selected ask the participant to tell you:
      i) *What's in the picture?*
      ii) *Why did they take the picture?* (explore any special meaning that the objects in the picture may have or if there are any stories / memories associated to this picture)
      iii) *Why did they select this picture, what makes it different from the other pictures they took?*
   c) Proceed in the same way with the other two topics: benefits and challenges.
   d) Now, taking into consideration **<u>all the activities-related pictures</u>** the participant has taken, ask her the following:
      Imagine that, of all the pictures you took, we will show ONE picture to others outside of your community to talk about fisheries activities in your village. Which one would you choose?

      Once the participant picked a picture, proceed to ask the following questions:

- *What is in the picture?*
- *Why did they take the picture?* (explore any special meaning that the objects in the picture may have or if there are any stories / memories associated to this picture)
- *Why did they select this picture? what makes it different from the other pictures?*
- *What would they like to tell to others with this picture?* What message do they want to transmit?
- *Why would it be important to give this message to others?*

3. Reflect on conclusions and finalise any comments.
4. Thank participants for their time and inform them that you will return in a weeks' time to validate findings and return the photographs.

D. OUTCOMES, VALIDATION and DISSEMINATION (STEPS 6, 7, 8)

FINAL PHOTOVOICE GROUP SESSION

The aims of this final Photovoice group session are to:

- Share the meanings and stories behind the stories and to ensure that participant voices are accurately captured and represented;
- Discuss outcomes of the project and dissemination activities;
- Capture group perspective on the Photovoice experience.

Before we kindly begin the session, are you happy for me to record the session via a Dictaphone?

1. PARTICIPANT CHECKING/REFLECTION:

a) During this exercise, all participants will have the opportunity to share their main messages captured during the discussion over 1-2 weeks ago. Each participant will also be able to ensure that the key messages captured are correct and specify whether you would like to add any further comments.
b) To begin the exercise, we have printed out all pre-selected images captured for topic 1, 2, 3 and overall for each participant. Please take the time to review these photographs in front of you.
c) We will now relay the key messages captured for these images one-by-one for each participant. Participant 1: core activity and message; core benefit and message; core challenge and message; overall core message. Are there any amendments or further comments?
d) REPEAT FOR ALL PARTICIPANTS.
e) AT END OF INDIVIDUAL DISCUSSION ASK THE GROUP WHETHER THEY HAD ANY FURTHER GROUP PERSPECTIVES ON 1) ACTIVITIES; 2) BENEFITS; 3) CHALLENGES.

2. DISSEMINATION ACTIVITIES

a) We would now like to ask all participants whether you have any wishes for how the findings will be disseminated. This will be an opportunity for you to think about how you want to share your pictures and stories with others in the community and beyond.
b) To begin, we would like to know who you may want to hear your stories? Who do you want to be your target audient- other fisher communities, stakeholders such as the Department of Fisheries, international organisations and other communities world-wise?

c) Secondly, how would you like your stories and pictures to be shared with the target audience? public exhibition, international academic conferences, online.

d) As a student, I would like to disseminate your findings via a public exhibition and at international conferences. Would you be happy for me to display these photographs in front of you along with the messages captured?

3. REFLECTION ON PV PROCESS

a) We would like to finish this session by asking for group feedback on your experience of participating in the project. This information will be greatly appreciated and will help assess the value of using photography in research projects.

- *What has been your overall experience participating in this project?*
- Was taking photographs a significant or meaningful part of your experience?
- Has your understanding of yourself and fishing changed throughout this project?
- Do you feel like you have gained confidence and/or comfort by participating?
- What has been the community response to the Photovoice project? Have you seen any changes (in attitudes or action)?
- Do you feel like your voice and story was heard in this project? Why or why not?
- How would you change this project if you were to participate again?
- Is there anything else you would like to add?
